# Prospective Clinical Trial on Expanding the Eligibility Criteria for Targeted Axillary Dissection after Neoadjuvant Systemic Therapy in Breast Cancers with Advanced Nodal Disease

**DOI:** 10.1245/s10434-026-20132-2

**Published:** 2026-08-28

**Authors:** A. J. T. Lee, B. H. H. Cheung, D. T. K. Suen, A. Kwong

**Affiliations:** Division of Breast Surgery, Department of Surgery, Daniel CK Yu Professor in Breast Cancer Research, Queen Mary Hospital, The University of Hong Kong, Hong Kong SAR, China

**Keywords:** Breast cancer, Targeted axillary dissection (TAD), Diagnostic accuracy, False negative rate (FNR), Neoadjuvant chemotherapy (NAC), Higher nodal burden, Axillary dissection (AD), Pathological complete response (pCR)

## Abstract

**Background:**

Targeted axillary dissection (TAD) is an established axillary restaging approach in breast cancer after neoadjuvant chemotherapy (NAC), but evidence to date has focused largely on clinical N1 disease with limited nodal involvement. For higher nodal burden patients, axillary dissection (AD) is still frequently performed despite high axillary pathologic complete response (pCR) rates. We hypothesized that TAD is noninferior to AD for nodal staging in patients with advanced nodal disease.

**Patients and Methods:**

Patients with higher nodal burden breast cancer planned for NAC followed by curative surgery were included. Higher nodal burden was defined as > 3 metastatic nodes up to clinical N3 stage. Patients underwent TAD followed by AD during the same operation. The primary outcome was the false negative rate (FNR) of TAD. Secondary outcomes included clipped node detection rate, concordance between clipped and sentinel nodes, and axillary pCR rates.

**Results:**

A total of 65 patients were prospectively recruited from February 2023 to October 2025; 10 withdrew, leaving 55 for analysis. The technical success rate of TAD was 96%, with clipped node detection rate of 100% and sentinel node detection rate of 96%. The clipped node was identified as an additional sentinel node in 68%. Axillary pCR occurred in 49%. In non-pCR patients, TAD demonstrated a FNR of 0%.

**Conclusions:**

In clinical N1 (> 3 nodes), N2, and N3 patients, a 0% FNR suggests TAD is highly accurate for detecting residual axillary disease in the setting of higher nodal burden. These results support expanding TAD eligibility in appropriately selected patients to potentially reduce AD and its morbidities.

Increase in axillary pathological complete response (pCR) after neoadjuvant systemic therapy in patients with clinically node-positive breast cancer has paved the way toward less invasive axillary treatment strategies such as targeted axillary dissection (TAD). Traditional axillary dissection (AD) subjects patients to significant morbidities, often leading to lifelong functional impairment. Over the years, accumulating evidence has suggested that axillary recurrence and overall survival are dictated predominantly by tumor biology and systemic therapy, rather than by the extent of nodal clearance.^1^ Consequently, the main purpose of axillary surgery after chemotherapy is to accurately restage the axilla to guide subsequent systemic treatment decisions.

Sentinel lymph node biopsy was initially adopted as a restaging tool; however, it encountered high false negative rates exceeding 10% in the ACOSOG Z1071 and SENTINA trials.^2,3^ The TAD technique was later introduced, combining sentinel lymph node biopsy with removal of a previously marked metastatic lymph node. By ensuring excision of the most suspicious and biopsy-proven metastatic node, TAD demonstrated superior diagnostic accuracy to sentinel lymph node biopsy alone, with reported false negative rates as low as 2–7%.^4–7^ While AD remains the gold standard for nodal staging, in patients who attain nodal pCR, a successful TAD procedure may allow for omission of formal AD, thereby reducing treatment-related morbidities without compromising staging accuracy.

At present, TAD is selectively applied to patients with breast cancer with a low nodal burden, typically clinical N1 disease with three or fewer metastatic lymph nodes, as most available data arise from cohorts with limited nodal involvement.^4,6,7^ In patients with more advanced nodal disease, the diagnostic performance and safety of TAD have not been properly addressed. Reflecting this uncertainty, major breast cancer guidelines including the National Comprehensive Cancer Network (NCCN),^8^ European Society for Medical Oncology (ESMO),^9^ and the German Gynecological Oncology Working group (AGO)^10^ have incorporated TAD in the workflow management of node-positive breast cancers that show good clinical nodal response to neoadjuvant chemotherapy, but the recommendation for patients with a higher nodal burden remains less established due to limited supporting evidence.

The present study aims to address this knowledge gap by evaluating diagnostic accuracy of TAD in axillary restaging after neoadjuvant chemotherapy, with a specific focus on patients presenting with a higher nodal burden.

## Patients and Methods

### Study Design

This was a prospective, single-center, cohort study conducted at a tertiary breast cancer center in Hong Kong from February 2023 to April 2026. We hypothesized that TAD is noninferior to conventional axillary dissection with regard to nodal staging in breast cancer patients with higher nodal burden—more than 3 pathological nodes. The primary objective of this study was to evaluate the accuracy of TAD in terms of the false negative rate (FNR) and negative predictive value (NPV). The secondary objectives were to determine the success rate of the clipped node removal, the concordance between the clipped node and the sentinel node status, and the rate of nodal pathological complete response. Ethical approval by the institutional review board of the University of Hong Kong/Hospital Authority (no. HKU/HA HKW UW 23-013) was obtained prior to commencement of this study. Written informed consent was obtained from all enrolled patients.

### Patient Selection

All female patients aged 18 years or older with clinically node-positive invasive carcinoma of the breast planned for neoadjuvant chemotherapy followed by curative surgery were screened for eligibility. The nodal involvement had to be histologically proven by fine needle aspiration or core biopsy. Higher nodal burden is defined by clinical N1 with more than 3 nodes, N2, or N3. Patients with distant metastasis, inflammatory breast cancer, previous ipsilateral axillary surgery, plan for neoadjuvant endocrine therapy without chemotherapy, and contraindications for superparamagnetic iron oxide (SPIO) injection, including allergy to iron or dextran compounds, and pacemaker or metallic devices implants in the chest wall, were excluded. In addition, pregnant, breast-feeding, or patients with mental incompetence were also excluded.

### Pretreatment Evaluation

All patients received imaging with mammogram and ultrasonography (USG) of the breast and axilla to determine tumor size and axillary staging. Axillary lymph nodes were considered suspicious on the basis of established sonographic features, including cortical thickening, loss of fatty hilum, and abnormal nodal morphology, as determined by the reporting radiologist. Pathological subtype was confirmed by core biopsy of the breast lesion and fine needle aspiration of the nodal disease. Metastatic workup was arranged for all patients according to institutional protocol with a positron emission tomography-computed tomography (PET-CT). The number of involved lymph nodes was determined on the basis of the number of hypermetabolic lymph nodes identified on PET-CT.

### Nodal Marking

Before commencement of neoadjuvant therapy, nodal marking with magnetic seed was performed under USG-guidance. When there were multiple lymph nodes, the previously proven metastatic node or the most abnormal node was targeted.

### Neoadjuvant Systemic Therapy

Neoadjuvant systemic therapy was administered in accordance with local and international guidelines by the treating oncology team.

### Posttreatment Evaluation

Interval clinical assessment of tumor response was performed every 6–8 weeks throughout the course of chemotherapy. After completion of neoadjuvant therapy, patients received mammographic and ultrasonographic reassessment of the breast and axilla. Presence of the visible seed and acoustic magnetic signal in the axilla was confirmed in clinic using USG and hand-held magnetometer, respectively, before operation.

### Study Procedure and Pathological Evaluation

Patients who had radiological complete or partial response after neoadjuvant systemic therapy received surgery of the breast and the axilla in the same session. For the axillary surgery, all patients underwent TAD followed by conventional axillary dissection. All procedures were performed by three surgeons experienced in TAD with standardized dual-tracer protocol—SPIO and blue dye, in addition to previous magnetic seed localization. TAD was defined by excision of clipped node along with sentinel lymph node biopsy. At least one sentinel lymph node should be excised in addition to the clipped node. Excision of clipped node was guided by the magnetometer, and presence of the magnetic seed was confirmed visually with intraoperative specimen mammogram. If either the clipped node or sentinel node could not be identified, this was considered failure of TAD. The clipped node was also a sentinel lymph node if it appeared blue or brown. Pathology of the TAD specimen and the subsequent axillary dissection specimen was performed using hematoxylin and eosin (H&E) staining, and the results were assessed and compared to determine diagnostic performance.

### Statistical Analysis

A prospective consecutive sampling approach was planned for sample size calculation. All eligible patients attending the breast oncology clinic during the prespecified recruitment period were screened in accordance with the inclusion and exclusion criteria. On the basis of local clinical data prior to study initiation, an estimated 20–30 patients per year were expected to meet eligibility criteria, yielding a target sample size of approximately 60 patients over the recruitment period. As this was an exploratory single-center study, the proposed sample size was determined mainly by anticipated case availability and practicality to ensure a sufficient number of node-positive patients for a robust estimation of FNR, rather than for formal hypothesis testing.

A false negative result occurred when TAD identified no residual disease despite metastatic nodes being present in the axillary dissection specimen. FNR was defined as the number of false negative results divided by the total number of patients with positive nodes. NPV was defined as the number of true negative results divided by the total number of patients with negative specimens. Data was prospectively collected and entered into a database. Statistical analysis was performed with SPSS statistics. Chi-squared test or Fisher’s exact test was used for categorical variables. Student’s *t*-test or Mann–Whitney *U* test was used for quantitative variables. *p*-Value of less than 0.05 was considered statistically significant. The 95% confidence intervals were calculated using the Wilson score interval.

## Results

**Fig. 1:**
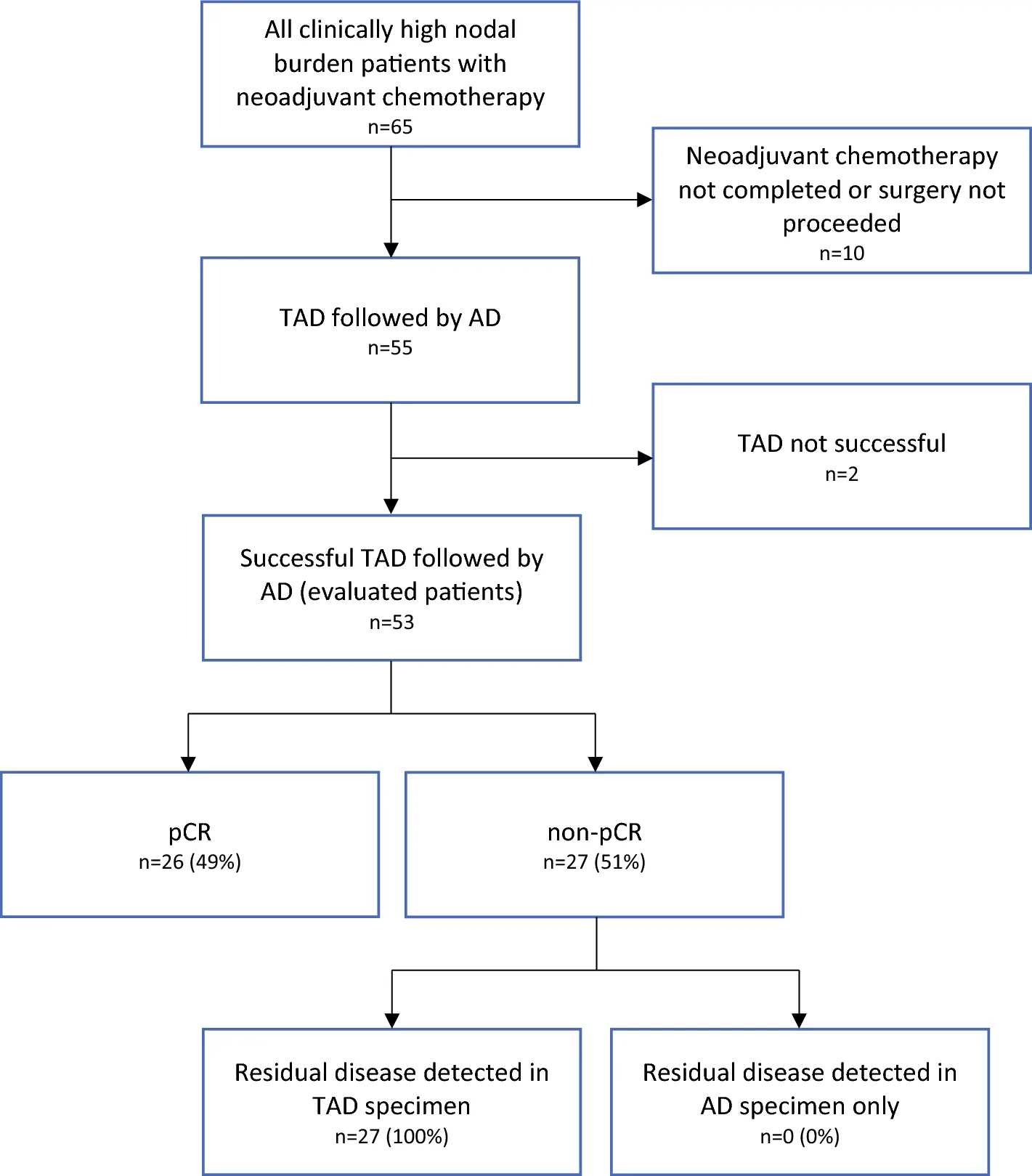
Flow diagram of patient recruitment and targeted axillary dissection (TAD) outcomes

A total of 65 patients were recruited from February 2023 to October 2025. All patients were Asian, comprising 80% Chinese, 18% Filipino, and 2% Indonesian; 10 were withdrawn due to relocation of treatment institution (5 out of 10), intolerance to chemotherapy (3 out of 10), poor response to chemotherapy (1 out of 10), and emigration (1 out of 10). The remaining 55 patients proceeded to receive the study procedure after neoadjuvant systemic therapy, as shown in Fig. 1. Patient and tumor characteristics are presented in Table 1. Median age was 58 (range 33–77) years. Most patients (64%) had clinical T2 staging with a median tumor size of 3.8 cm, and 53% percent of patients had clinical N2 stage or higher. Median number of pathological lymph nodes on pretreatment imaging was 4. The tumor subtype distribution comprised 20% triple-negative, 49% HER2-positive, and 31% hormone receptor (HR)-positive/HER2-negative.

**Table 1:** *Patient and tumor characteristics* (n = *55*)

Age, years (median, range)	58 (33–77)
Ethnicity	
Chinese	44 (80%)
Filipino	10 (18%)
Indonesian	1 (2%)
Clinical tumor staging before systemic therapy	
T1	3 (5%)
T2	35 (64%)
T3	15 (27%)
T4	2 (4%)
Tumor size, cm (median, range)	3.8 (1.1–10.2)
Clinical lymph node staging before systemic therapy	
N1	26 (47%)
N2	7 (13%)
N3	22 (40%)
N3a	2 (9%)
N3b	17 (77%)
N3c	3 (14%)
Median size of largest axillary lymph node, cm (range)	1.9 (0.7–55)
Number of pathological lymph nodes on imaging before neoadjuvant systemic therapy (median, range)	4 (2–10)
Histology	
Invasive ductal carcinoma	53 (96%)
Invasive lobular carcinoma	0 (0%)
Other (papillary)	2 (4%)
Modified Bloom–Richardson score	
Grade 1	0 (0%)
Grade 2	9 (16%)
Grade 3	15 (27%)
Ki67%	
< 20%	2 (4%)
≥ 20%	49 (89%)
Tumor subtype	
HR + HER2−	17 (31%)
HR + HER2+	19 (29%)
HR − HER2+	11 (20%)
HR − HER2−	11 (20%)
Breast surgery	
BCS	11 (20%)
Mastectomy	44 (80%)
Axillary pCR	
Yes	28 (51%)
No	27 (49%)

*HR* hormone receptor, *HER2* human epidermal growth factor receptor-2, *BCS* breast conserving surgery

### Study Procedure

In 53 out of 55 patients (96%) the clipped node and at least one additional sentinel lymph node was removed, signifying a successful TAD (Table 2). In the two patients with a failed TAD procedure, clipped node was found but no sentinel nodes were identified. Hence the overall clipped node identification rate was 100% (55 out of 55) while the sentinel node identification rate was 96% (53 out of 55). Both of these patients with an unsuccessful TAD achieved axillary pCR, with no residual disease identified in the axillary dissection specimen.

**Table 2:** Intraoperative data

Time taken to perform TAD, min (median, range)	25 (10–90)
Clipped node identification (%)	55/55 (100%)
Sentinel node identification (%)	53/55 (96%)
TAD success (%)	53/55 (96%)
Number of sentinel nodes retrieved (median, range)	3 (1–9)
Concordance of clipped node and sentinel node status (%)	36/53 (68%)
Brown SPIO uptake	17/36 (47%)
Blue dye uptake	2/36 (6%)
Both brown and blue uptake	17/36 (47%)

In the 53 patients with a successful TAD, the median number of sentinel nodes retrieved was 3 (range 1–9). The clipped node was concordant with sentinel node status in 68% (36 out of 53) patients, with 6% blue, 47% brown, and 47% both blue and brown. In 32% (17 out of 53) patients, the clipped node was not a sentinel node. No factors associated with clipped node and sentinel node discordance were identified on subgroup analysis (Table 3). The median time taken to perform TAD was 25 (range 10–90) min.

**Table 3:** Factors associated with clipped node and sentinel node concordance

	Overall number (%)	Clipped node concordant with sentinel node status (%)	Clipped node discordant with sentinel node status (%)	*p*-Value
Initial size of largest node (mm)				0.066
≤ 10	9 (17%)	8 (89%)	1 (11%)	
> 10–20	24 (45%)	17 (71%)	7 (29%)	
> 20	20 (38%)	11 (55%)	9 (45%)	
Initial number of suspicious nodes				0.168
≤ 4	27 (51%)	16 (59%)	11 (41%)	
> 4	26 (49%)	20 (77%)	6 (23%)	
Clinical N stage				0.518
1	26 (49%)	17 (65%)	9 (35%)	
2	7 (13%)	4 (57%)	3 (43%)	
3	20 (38%)	15 (75%)	5 (25%)	
HR+/HER2−				0.122
No	36 (68%)	22 (61%)	14 (39%)	
Yes	17 (32%)	14 (82%)	3 (18%)	
HER2				0.243
Negative	28 (53%)	21 (75%)	7 (25%)	
Positive	25 (47%)	15 (60%)	10 (40%)	
Triple negative				0.732
No	42 (79%)	29 (69%)	13 (31%)	
Yes	11 (21%)	7 (64%)	4 (36%)	
Clinical nodal status after NAC on radiological exam*				0.071
Partial response	38 (72%)	28 (74%)	10 (26%)	
Complete response	13 (25%)	6 (46%)	7 (54%)	

^*^Clinical nodal status after NAC was based on reassessment with mammogram, ultrasonography, and positron emission tomography-computed tomography

*HR* hormone receptor, *HER2* human epidermal growth factor receptor 2, *NAC* neoadjuvant chemotherapy

### Diagnostic Accuracy of TAD

Axillary pCR rate was 49% (26 out of 53 patients), with 64% of those being HER2-enriched disease and 25% triple-negative disease. In the remaining 51% (27 out of 53 patients) with axillary non-pCR, the residual nodal disease was identified by TAD (in either the clipped node or sentinel nodes), yielding a FNR of 0% (95% CI 0–0.13) and NPV of 100% (95% CI 0.87–1.0) as presented in Table 4. In 14 of 27 patients (52%) only the clipped node was positive, while in 5 of 27 patients (19%), only the sentinel node(s) were positive. After incorporating clipped nodes that were also sentinel-concordant, the FNR of sentinel lymph node biopsy alone was found to be 11.1% (Table 5), while for clipped node excision alone it was 18.5% (Table 6). In the axillary dissection specimen after those with a positive TAD, additional pathological nodes were found in 63% patients, with a median of 2 nodes (range 1–7).

**Table 4:** Diagnostic accuracy of TAD

	Additional nodes (axillary dissection)		
	Positive	Negative	Total no.
TAD positive	17	10	27
TAD negative	0	26	26
Total no.	17	36	53

FNR: 0/27 = 0% (95% CI 0–0.13)

NPV: 26/26 = 100% (95% CI 0.87–1.0)

Overall diagnostic accuracy: 53/53 = 100% (95% CI 0.93–1.0)

**Table 5:** Diagnostic accuracy of sentinel lymph node biopsy

	Additional nodes (clipped node or axillary dissection)		
	Positive	Negative	Total no.
Sentinel node positive	15	9	24
Sentinel node negative	3	26	29
Total no.	18	35	53

FNR: 3/(24 + 3) = 11.1% (95% CI 0.04–0.28)

NPV: 26/(26 + 3) = 89.7.% (95% CI 0.74–0.96)

Overall diagnostic accuracy: (24 + 26)/53 = 94.3% (95% CI 0.85–0.98)

**Table 6:** Diagnostic accuracy of clipped node excision

	Additional nodes (sentinel node or axillary dissection)		
	Positive	Negative	Total no.
Clipped node positive	17	5	22
Clipped node negative	5	26	31
Total no.	22	31	53

FNR: 5/(22 + 5) = 18.5% (95% CI 0.08–0.37)

NPV: 26/(26 + 5) = 83.9% (95% CI 0.67–0.93)

Overall diagnostic accuracy: (22 + 26)/53 = 90.6% (95% CI 0.80–0.96)

## Discussion

Literature has consistently shown that axillary pCR can be achieved in up to 60% of clinically node-positive patients,^1^ particularly in the HER2-positive and the triple-negative subtypes. In this setting, de-escalated axillary surgical approaches have been increasingly adopted to reduce surgical morbidity. The uncertainty remains in patients with a higher nodal burden—whether de-escalated axillary surgery results in higher false negative rates, which can jeopardize diagnostic and prognostic outcomes. One of the largest landmark trials on TAD was only able to include 28% of patients with more than three pathological nodes in their study.^4^ A more recent multicenter prospective trial noted higher false negative results in cN2 versus cN1 patients, although further analysis was limited by a small number of patients in the cN2 subgroup.^6^ To our knowledge, this is the first paper to focus on patients with higher nodal burden while demonstrating feasibility and safety of TAD after neoadjuvant systemic therapy.

All nodal marking was performed with magnetic seed in this study, which relieves the patient from a second perioperative localizing procedure. During the course of neoadjuvant chemotherapy, visualization of clip with bedside USG is recommended to help detect potential clip migration. Of note, medial clip migration to the intercostal muscles was observed during formal axillary dissection in a patient excluded from analysis due to intolerance and early termination of chemotherapy.  Amongst the evaluated patients, the clipped node identification rate was 100% using intraoperative auditory signal, with additional confirmation by specimen mammogram. It has been suggested that clipping and removing all metastatic nodes without performing sentinel lymph node biopsy may increase the accuracy of axillary staging;^11^ however, findings from this trial demonstrate that clipping only the most representative pathological node, even in advanced nodal disease, is sufficient to achieve high diagnostic accuracy. Clipping and removing all pathological nodes across all patients may introduce additional practical challenges.

In the two patients with an unsuccessful TAD, the main issue was failure to identify any additional sentinel lymph nodes. Both patients had cT2N3b HER2-positive breast cancer and achieved pCR following neoadjuvant systemic therapy. In the first patient, the clipped node was the only sentinel node and showed both blue and brown staining. In the second patient, the clipped node did not correspond to a sentinel lymph node. One possible explanation for failed sentinel node localization is that the marked treatment response in a heavily involved axilla may lead to increased fibrosis of lymphatic channels and impaired tracer migration. Consistent with this, lower sentinel lymph node identification rates in patients converted from clinically node-positive to node-negative after NAC have been reported in large trials, such as the SENTINA study.^3^ Previously, the MARI study reported a 7% false negative rate with clipped node removal alone, without retrieving any other additional sentinel nodes.^5^ However, most patients in that study had cN1 disease, limiting applicability to our patients with a higher nodal burden.

In this study, clipped node status was concordant with sentinel node status in 68% of patients, consistent with reported figures in literature ranging from 65 to 77%.^4,6,7^ Subgroup analysis did not identify any factors that were associated with clipped node and sentinel node discordance, contrary to findings reported by Caudle et al.^4^ The FNR for clipped node excision alone was 19% in our patient cohort, higher than previously reported rates in studies predominantly involving cN1 patients.^5^ This likely reflects the challenge of accurately identifying the previously histologically proven or most representative node in the presence of multiple abnormal lymph nodes in patients with heavy nodal involvement, thereby increasing the clipped node FNR. Nonetheless, once clipped node excision and sentinel lymph node biopsy were performed together, the overall FNR rate was exceptionally low, supporting the diagnostic superiority of the TAD approach over clipped node excision or sentinel node biopsy alone, particularly in patients with higher nodal burden. In this cohort, harvesting two lymph nodes including the clipped node was sufficient to achieve an acceptable FNR. These findings also bear important implications for adjuvant treatment planning, including escalation or de-escalation of subsequent treatment intensity based on residual nodal burden and overall pathologic response. The recent NSABP B-51 trial, for example, requires a minimum of two pathologically negative sentinel lymph nodes to allow for safe omission of regional nodal irradiation in the setting of pCR.^12^

One limitation of this single-center study is the relatively small sample size which produced wide confidence intervals for the false negative rate. Larger studies are therefore recommended to provide more precise estimates. A second limitation relates to assessment of clipped node to sentinel node concordance. In this study, dual tracers—blue dye and SPIO—were used instead of conventional radioisotope. The choice of SPIO is well supported by prior randomized studies demonstrating its efficacy for sentinel lymph node detection.^13^ However, acoustic signal from the magnetic seed in the clipped node may overpower the SPIO signal; as a result, sentinel node concordance was determined primarily by visual assessment of blue or brown staining. Visual assessment is inherently subjective and may be influenced by factors such as perinodal adipose tissue thickness or specimen bulk, potentially leading to underestimation of true concordance. Ideally, applying radioisotope with an acoustic signal distinct to SPIO would allow for more objective concordance assessments. Finally, the generalizability of our findings may be limited by the single-center design, involvement of only three surgeons, and a homogeneous study population comprising exclusively Asian patients.

In conclusion, this study demonstrates that TAD is a feasible and highly accurate method for detecting residual axillary disease, with a 0% FNR even in patients with up to cN3 disease after neoadjuvant chemotherapy. With approximately half of patients achieving axillary pCR, TAD offers a substantial opportunity to avoid AD in a large proportion of initially node-positive patients. Nevertheless, larger multicenter studies are needed to validate these findings and to further establish the oncological safety of AD omission to support the potential expansion of TAD eligibility criteria.
